# Predicting neuroticism with open-ended response using natural language processing

**DOI:** 10.3389/fpsyt.2024.1437569

**Published:** 2024-08-01

**Authors:** Seowon Yoon, Jihee Jang, Gaeun Son, Soohyun Park, Jueun Hwang, Joon Yeon Choeh, Kee-Hong Choi

**Affiliations:** ^1^ School of Psychology, Korea University, Seoul, Republic of Korea; ^2^ KU Mind Health Institute, Korea University, Seoul, Republic of Korea; ^3^ Department of Software, Sejong University, Seoul, Republic of Korea

**Keywords:** personality prediction, natural language processing, language analysis, neuroticism, open-ended questions, computational personality assessment

## Abstract

**Introduction:**

With rapid advancements in natural language processing (NLP), predicting personality using this technology has become a significant research interest. In personality prediction, exploring appropriate questions that elicit natural language is particularly important because questions determine the context of responses. This study aimed to predict levels of neuroticism—a core psychological trait known to predict various psychological outcomes—using responses to a series of open-ended questions developed based on the five-factor model of personality. This study examined the model’s accuracy and explored the influence of item content in predicting neuroticism.

**Methods:**

A total of 425 Korean adults were recruited and responded to 18 open-ended questions about their personalities, along with the measurement of the Five-Factor Model traits. In total, 30,576 Korean sentences were collected. To develop the prediction models, the pre-trained language model KoBERT was used. Accuracy, F1 Score, Precision, and Recall were calculated as evaluation metrics.

**Results:**

The results showed that items inquiring about social comparison, unintended harm, and negative feelings performed better in predicting neuroticism than other items. For predicting depressivity, items related to negative feelings, social comparison, and emotions showed superior performance. For dependency, items related to unintended harm, social dominance, and negative feelings were the most predictive.

**Discussion:**

We identified items that performed better at neuroticism prediction than others. Prediction models developed based on open-ended questions that theoretically aligned with neuroticism exhibited superior predictive performance.

## 1 Introduction

Most personality measures rely on self-reported questionnaires (1, 2). While providing practicality, self-report questionnaires have been criticized for their length, limited information, high face validity, and response bias (3). As psychology integrates artificial intelligence and machine learning, research has shown that various types of data can predict specific psychological constructs. Personality researchers have adopted computational science as an alternative to self-reported measures (4, 5). Computational personality assessment (CPA) uses technology at any stage to estimate personality (6). This approach includes automated personality assessment, machine-learning personality assessment, and language-based personality assessment (LPA). Language data, digital footprints, and mobile sensing are frequently used (4, 7). Computational personality studies with various types of data have been thoroughly reviewed by Stachl et al. (6) and Bleidorn and Hopwood (8) in their recent works.

Despite promising results, CPA studies often fail to meaningfully translate predictive studies into psychological theories (1, 8, 9). As CPA lies at the intersection of two distinct disciplines—psychology and computer science—CPA studies must overcome notable differences in norms and a considerable lack of connection between the two fields (10). CPA studies conducted in the field of computer science have improved personality prediction models, but they often only cite psychological studies for using personality inventories rather than discussing psychological implications (6). However, psychology has shown less interest in CPA, which is further hampered by modeling practices’ increasing complexity (2, 11).

CPA studies using language data often employ text data from social media platforms (4, 12, 13). Previously, myPersonality was a popular resource for obtaining personality data because it contained the personality information of 4.5 million Facebook users (14). However, researchers are exploring alternative language data tagged with personality information since myPersonality dataset is no more publicly available. As a result, recent LPA studies have frequently used language datasets with the Myers–Briggs Type Indicator (15), despite its questionable psychometric validity (16). Because it is challenging to acquire language datasets for machine learning that incorporate valid personality information, personality labels are often assigned by researchers through annotation instead of using validated personality inventories, which are considered the ground truth for supervised learning (12, 13). Instead of measuring personality through valid measure, this annotation procedure reduces personality label validity.

Another concern in CPA is content validity, primarily due to its reliance on the validity of predictive methodologies (8, 17). Currently, our understanding of what and how the computational models measure remains limited (18). For instance, facial image personality prediction algorithms may focus on background brightness rather than actual image content or facial features (6). Given that facial expressions often reveal momentary emotional states rather than stable constructs like personality (19), it is important to compare data-driven results with psychological theories.

Computational personality research benefits from computational science norms, especially in acquiring large datasets for machine-learning approaches. To advance computational personality science and provide meaningful psychological insights, these limitations must be addressed from a psychological perspective (8, 9, 20). Limitations may include but are not limited to, the validity of personality labels, practicability, the context of language use, content validity, and employing readily accessible data without hypotheses (9, 17). One of the persistent issues in the field of computational science applied to psychological and personality assessments is the difficulty in understanding what machine learning algorithms actually measure (18). Bleidorn and Hopwood (8), citing Tellegen (21), argue that it is crucial to distinguish whether computational personality assessment measures personality itself or something related to it, no matter how trivial this distinction may seem. As discussed in the study by Segalin et al. (22) on personality prediction using images, classification algorithms may learn and produce results based on noise that humans neither intend nor expect. For instance, extroverted individuals tend to take their selfies in brighter lighting, and the algorithm might rely heavily on the background color rather than the face or expression for classification. More importantly, while facial expressions or features may predict momentary states, they are insufficient for predicting stable and enduring personality traits (19). Incorporating theoretical aspects, such as content validity, alongside insights derived from modern computational techniques, will enhance the validity of language-based personality assessments (20).

Another significant issue with previous literature is that social media data present substantial limitations regarding representativeness and bias, and they have not been validated in contexts outside of social media usage. Recent studies suggest that data obtained from social media often lacks representativeness compared to samples collected using traditional methods (2, 23–26). Moreover, Boyd and Schwartz (9) expressed concerns about the use of easily accessible corpus data without hypotheses. Data collected without hypotheses and design rarely reflect the constructs being measured. For instance, it is unlikely to obtain meaningful information about empathy from student assignments submitted as coursework (9). Additionally, since social media is a platform for self-presentation, it is likely to contain data biased towards traits such as extraversion and attention-seeking. Certain personality traits may be specific to individuals who use social media extensively. For these reasons, Zunic et al. (27) emphasized that, particularly in health-related fields, it is essential to construct datasets with concrete objectives and hypotheses prospectively, rather than relying on pre-existing data.

Using open-ended questions based on personality theory can serve as a valuable solution toward incorporating the norms of both disciplines. By offloading and integrating their expertise into open-ended questions, psychologists can ensure content validity (20). The use of open-ended questions is recommendable in the current language analysis paradigm (28). Traditional self-report inventories have limitations because researchers frame the questions and response options, and the forced-choice format limits participants’ responses (28, 29). The use of open-ended questions enables respondents to reveal various aspects of themselves in a relatively less constrained manner (30). Answers to open-ended questions are more likely to contain a multifaceted, comprehensive portrayal of respondents, including the order, interactions, and context of events (28). Unlike self-report questionnaires that limit responses to predefined options, text responses can capture a broader range of emotions, thoughts, and behaviors, providing a more comprehensive picture of an individual’s psychological state (31). Additionally, verbal expressions have been shown to consistently provide stable predictions regarding personality. Park et al. (4) reported that the test-retest reliability of language-based personality assessments was robust, with an average reliability of 0.70. Furthermore, open-ended questions serve as a framework for respondents to reveal their personalities in clinical or research settings because language varies according to context, and responses are significantly influenced by the questions posed. Previous CPA studies have not provided practical guidelines for implementation in clinical or research contexts.

In this study, we aimed to predict individuals’ personality traits by using natural language processing (NLP) techniques to analyze their responses to open-ended questions. The current study focused on neuroticism, among several other personality traits. Neuroticism is one of the most salient predictors of personality traits (32). Previous studies have highlighted the predictive aspects of neuroticism due to its strong association with mental disorders, physical health, public health, and psychosocial functioning (33–35).

In the present study, we constructed models for each of the 18 open-ended questions, identifying questions that most effectively predicted neuroticism and its two lower-order dimensions: depressivity and dependency. Previous studies have recommended avoiding readily available language data (9, 36), yet no study has explored the benefits of a theory-based approach that includes data collected with hypotheses in the field of LPAs. With open-ended questions specifically designed to assess each personality trait, the current study provided unique information on the influence of item content on predictive aspects. This study aimed to investigate the following research questions: 1) What level of predictive performance can be achieved when constructing prediction models using responses to open-ended questions based on a relatively small sample size of 425 individuals? 2) Which questions are particularly effective in predicting neuroticism? and 3) Compared with other models, does a prediction model using questions eliciting responses with content related to neuroticism demonstrate superior performance?

Detailed study procedure is listed in the protocol article; please refer to (37). The protocol includes an overview of the aim, design, data collection plans, measures, analysis plans, and anticipated results of the current study. The data have not been made available on a permanent third-party archive because our institutional review board ruled that we could not post the data; requests for the data and code can be sent to the corresponding author, but access to the raw data is limited to qualified researchers, especially for the verbal data. The materials used in these studies are widely available.

## 2 Materials and methods

### 2.1 Procedure

#### 2.1.1 Phase 1 (preliminary) study

The current study used open-ended questions specifically developed for LPA. In the preliminary stage (Phase 1), a multidisciplinary research team comprising psychologists, experts in human resources, and psychometricians created an item pool consisting of 66 open-ended questions. Each question aligned with a specific personality trait domain or facet in the five-factor model of personality (FFM) and an alternative model of personality disorder in the Diagnostic and Statistical Manual of Mental Disorders introduced by American Psychiatric Association.

From this initial item pool, 29 open-ended items were selected through expert review. A pilot study was then conducted with the selected 29 items among 57 Korean adults, all of whom responded to open-ended questions and completed a self-reported personality inventory. Information theory was applied to identify the entropy of linguistic expressions that showed a strong association with specific personality traits, and Latent Dirichlet Allocation was used for exploratory purposes. An external expert committee of psychologists reviewed 29 open-ended items and provided feedback regarding content validity, differential validity, clarity of expression, and social desirability bias. Through this combined computational analysis and expert committee review, the 29 preliminary items were revised and condensed into a final set of 18 open-ended questions for the LPA (**Supplementary Table 1**).

#### 2.1.2 Phase 2 study

In the current study (Phase 2), which began in November 2021, we collected data from participants using the open-ended questions finalized during Phase 1. Responses to self-reported questionnaires were collected online, whereas responses to open-ended questions were gathered via one of three methods: video interviews, chat interviews, or surveys. This study aimed to ensure the generalizability of the findings beyond online social media environments by collecting data from various language-use contexts. The video interviews were conducted over Zoom (https://zoom.us). The chat interviews were performed using an online counseling platform, Mindeep (https://mindeep.co.kr), while Qualtrics (https://qualtrics.com) was used for the essay format in the surveys. Video and chat interviews were conducted by four postgraduate students majoring in clinical psychology. The interview processes were standardized, involving identical questions presented in the same order. In the case of a need for further inquiry, a standardized question, “Could you please elaborate further?” was employed. The video interview was recorded and three researchers (SY, JJ, and GS) transcribed the responses question by question on an encrypted storage after the interview. The transcribed responses were processed the same as those received from the chat interviews and online surveys later. Finally, all the data were processed and analyzed with text feature.

### 2.2 Participants

This study used data collected from 425 newly recruited participants during the second phase of the research. Participants were recruited using convenience sampling, which included advertisements through a university-affiliated research institute, online community websites, and online job platforms. All Korean-speaking adults aged 19 years or older were eligible to participate in the study. Participants were asked to participate in either video or chat interviews and online surveys with given guidelines. Depending on the length of the individual’s responses, the interview could be lengthy, so full concentration during the interview was required. In addition, the individual had to be able to respond to the questions in the interview and the online survey by themselves, so they needed to have sufficient intellectual ability and Korean language skills to understand the given questions. Given this context, the exclusion criteria included severe mental illness with acute psychotic symptoms, a history of neurosurgery, intellectual disability, and an inability to communicate with the researchers. However, no participants were excluded based on the exclusion criteria, and all data from the 425 participants were included in the analysis. To avoid the biased distribution of the scores on personality measures and recruite participants with sufficiently high personality-related scores, we added a depression, anxiety, and personality screening phase prior to the interview and online survey phase.

Currently, guidelines for determining the appropriate sample size for computational language analysis when applying methodologies similar to those used in this study have not been established. In the field of computational studies, it has been reported that classification models can be built with a minimum of 80–560 labeled data points (38). Previous CPA studies that did not use social media data reported sample sizes ranging from 100 to 400 individuals (29, 39).

In the present study, 425 participants were recruited, all of whom provided demographic information such as sex, age, education, marital status, household income level, occupation, and history of psychiatric and neurological diagnoses during the second stage of the research. All participants voluntarily provided written informed consent, and their remuneration was 40,000 KRW (35 USD). This study was approved by the institutional review board of the university. Two hundred seventy participants were female (63.5%) and one hundred fifty-five were male (36.5%); Mean age of the sample was 31.4 years old (*SD* = 8.6 years) and mean education years were 15.4 years (*SD* = 1.8 years). The demographic characteristics of the participants are presented in **Table 1**.

**Table 1:** Demographics of Participants.

Category			Frequency	%
Age (Mean / SD)			31.4 (8.6)	
Years of Education (Mean / SD)			15.4 (1.8)	
Gender	Male		155	36.5
	Female		270	63.5
Monthly household income (Unit: $)	Less than 1,200		50	11.8
	1,200 to 2,500		91	21.4
	2,500 to 3,400		63	14.8
	3,400 to 4,300		64	15.1
	4,300 to 6,600		85	20.0
	More than 6,600		72	16.9
Marital status	Single		338	79.5
	Married		84	19.8
	Divorced/Widowed		3	0.7
Student sample	Undergraduate participants		155	36.5
Psychiatric diagnosis	Individuals with any history of psychiatric diagnosis		61	14.4

*N* = 425.

### 2.3 Measures

The participants submitted their demographic information and responded to 18 open-ended questions (**Table 2**) for LPA. All participants completed a battery of questionnaires via the online survey platform Qualtrics (https://qualtrics.com) to measure their personality traits and other psychological constructs. The participants were instructed to provide a free response containing a minimum of three sentences with no prescribed upper limit. Of the 425 participants, 106 responded through video interviews, 139 through chat interviews, and 180 through essay-format surveys.

**Table 2:** Open-ended items for language-based personality assessment.

	Items
1	How do you want to spend your time for your routine daily hours?
2	Do you prefer a leading position in your work or interpersonal relationships? Or do you prefer to contribute from a less leading position?
3	What is the main reason for having difficulties in trusting your friends or associates?
4	What do you usually think and do when someone asks for help? Tell us how you feel and think when rejecting someone’s request
5	How do you usually handle tasks that need to be completed in a given schedule?
6	To what extent do you tend to achieve the standards or goals you set for yourself?
7	Do you frequently compare yourself to others? Please share about this.
8	How do you feel and think when you meet new kinds of experiences?
9	How would you feel if someone else noticed your feelings (joy, happiness, anxiety, anger, sadness)?
10	To what extent do you feel cautious or want to avoid interpersonal relationships?
11	How close do you want your relationships to be with others?
12	To what extent do you want to be recognized or treated by people?
13	How do you feel about being noticed or receiving attention from others?
14	How do you feel about someone who may be unintentionally hurt or harmed by you?
15	What is the main reason for having difficulties in doing things efficiently?
16	Tell me if there is something unusual in your actions or thoughts, or something that other people do not understand well.
17	Please share your negative feelings or thoughts if you have any.
18	Are you spontaneous and highly influenced by your mood? What do you do when you feel negative emotions?

#### 2.3.1 Bright and dark personality inventory

The current study used the Bright and Dark Personality Inventory (BDPI) (40) to assess participants’ neuroticism. The BDPI, which was developed based on the theoretical framework of the FFM (41), consists of 173 items rated on a 4-point Likert scale (ranging from 1 = strongly disagree to 4 = strongly agree). The BDPI was developed to comprehensively understand both personality dimensions from the Five-Factor Model of Personality and maladaptive personality dimensions, aligning with the alternative perspective suggested in the DSM-5 (Diagnostic and Statistical Manual of Mental Disorders 5), which proposes understanding personality not categorically but dimensionally (42). Maladaptive personality traits are closely related to the traits described by the FFM (43–45). For instance, negative affectivity (as opposed to emotional stability) in DSM-5 and ICD-11 is associated with neuroticism in the FFM, while detachment (as opposed to extraversion) corresponds to the maladaptive and extreme form of introversion (46, 47). The alternative model for personality disorders in DSM-5 can thus be seen as an extended model of the FFM (43–45). BDPI considers the traits of FFM and maladaptive personality traits belong to the same dimensional structure rather than being separate categories, as revealed by numerous studies (48–50). The BDPI measures and evaluates the multifaceted aspects of personality by integrating the traits of FFM maladaptive personality traits, allowing for a more detailed description of one’s personality.

The BDPI measures 10 personality traits, including five traits suggested by the FFM (extraversion, agreeableness, conscientiousness, openness, and emotional stability) and five maladaptive traits (detachment, antagonism, disinhibition, psychoticism, and neuroticism), each consisting of low-order dimensions (facets). As a result, the BDPI measures a total of 10 personality traits and 33 facets based on the theoretical framework of the FFM and DSM-5. BDPI assigns a score to each trait and facet, which is then converted into a *T*-score. Higher scores indicate a greater level of corresponding personality traits. The *T*-score of the BDPI was validated based on a stratified sample of 1,200 Korean adults, considering factors such as sex, age, education, and marital status. The *T*-score of high-order trait dimensions, such as neuroticism, was calculated as the weighted average of the facet scores (e.g., dependency and depressivity).

The BDPI has been psychometrically validated, including by the Item Response Theory, reporting adequate reliability and validity (40). Kim et al. (40) reported that Cronbach’s alpha coefficient was 0.92 for general personality scales and 0.96 for maladaptive personality scales. In the current study, Cronbach’s alpha coefficient was 0.89 for all items of the BDPI and 0.91 for neuroticism. The test-retest reliability of the BDPI was assessed with 114 participants, and satisfactory results were reported, with a Pearson’s coefficient of 0.84 and a Spearman’s coefficient of 0.81. The interval between the initial test and retest varied among participants, with a mean of 126 days (standard deviation (*SD*) = 81 days). The result of reliability analysis revealed that the BDPI demonstrated adequate level of internal consistency and test-retest reliability.

### 2.4 Language-based neuroticism prediction model

#### 2.4.1 A multi-class classification approach

To build the prediction model, we chose a three-class classification approach for predicting neuroticism. Given that the self-reported personality inventory BDPI calculates *T*-scores using a mean of 50 and a standard deviation of 15 for individuals’ neuroticism scores, adopting a regression approach would be a viable alternative. However, in the case of personality, most individuals do not exhibit extreme traits characteristic of personality disorders but rather converge near the mean value of a normal distribution. The subtle differences in language used at these levels necessitate a dataset comprising at least tens of thousands entries to develop a reliable regression model. This requirement has led many previous studies to adopted classification models to distinguish between high and low personality traits utilizing easily accessible social media data (4, 12, 13). To this end, we aimed to develop a prediction model based on verbal expressions that reflect the psychological constructs we intended to measure despite sacrificing some data size. This study aimed to better reflect the dimensionality of personality by constructing a more challenging multi-class classification model: below-average (*T* < 45), average (45 ≤ *T* < 55), and above-average (55 ≤ *T*) neuroticism. The groups consisted of 128, 134, and 163 participants, respectively.

#### 2.4.2 Input and output of the prediction model

The constructed model aimed to predict neuroticism levels (below-average, average, and above-average) based on responses to each open-ended question. One of the key decisions during this process was whether to set the input and output on a per-participant or per-sentence basis. If determined on a participant basis, all sentences from a participant’s response to a single question were compiled into a single input, and the output corresponded to the participant’s level of neuroticism. Recent pre-trained language models, such as BERT (Bidirectional Encoder Representations from Transformers), often exhibit improved contextual understanding when processing a coherent piece of text of suitable length (e.g., within 512 tokens) rather than treating each sentence separately. The output was determined on a per-participant basis because the primary goal of our study was to predict an individual’s personality rather than conduct sentiment analysis on a sentence-by-sentence basis. Consequently, each participant’s response to a single question was treated as one input, and the output was set as the individual’s neuroticism level. Using 18 open-ended questions (**Table 2**), we constructed 54 prediction models for neuroticism and its facets, dependency, and depressivity (18 questions × 3 indices).

#### 2.4.3 Fine-tuning of a pre-trained language model

First, we transcribed the responses to open-ended questions from the video interviews into a textual format. We did not conduct specific stop-word processing, as stop-word lists frequently include psychologically meaningful expressions, such as pronouns. Subsequently, embedding, which is a technique for representing text data as vectors of numbers, was implemented. In machine learning, it is necessary to convert text data into vector representations to facilitate their application in computational algorithms. Improved embedding techniques enhance the ability of the model to capture semantic relationships between words, consider context, and reduce computational demands, leading to improved performance and reduced training time.

A recent approach to NLP tasks involves the utilization of pre-trained language models with partial modifications to fit the needs of an individual downstream task. Models built solely on project data often result in performance degradation because of the restricted size of the dataset and their potential inability to effectively generalize to unseen data. Thus, current machine-learning models are rarely built from scratch. The application of transfer learning with pre-trained models facilitates the development of better models, even with relatively small datasets.

In the field of NLP, pre-trained language models, such as BERT and generative pre-trained transformers (GPT), are widely available. These pre-trained language models have been used for specific purposes in downstream tasks, with the process of training on downstream tasks using pre-trained models referred to as “transfer learning and fine-tuning.” In this study, we constructed a neuroticism prediction model by applying transfer learning and fine-tuning the widely used KoBERT pre-trained language model in Korea. KoBERT, a language model based on a transformer structure such as GPT or BERT, has been trained on Korean data from Wikipedia, including 5 million sentences and 54 million words, and has demonstrated good performance across various fields.

SentencePiece (51) is a universally applicable embedding tool that uses sentences obtained from participants as inputs for the KoBERT model. To predict the individuals’ neuroticism, we added a layer to the final output stage of KoBERT to perform this classification task. All the procedures were conducted using Python version 3.8.10.

#### 2.4.4 Training and validation process

**Figure 1:**
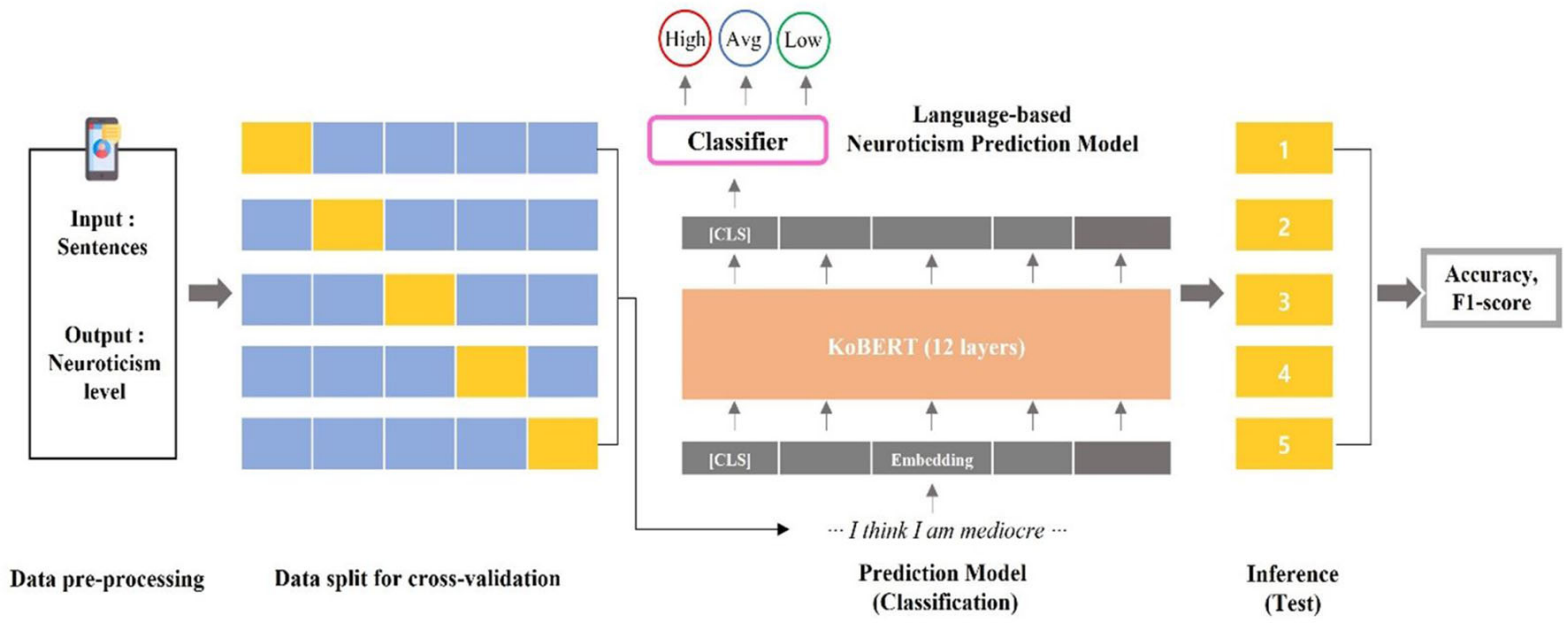
Process for training the neuroticism prediction model.

The current study employed a 5-fold cross-validation approach. The training–validation process was repeated five times; each time, a different section of dataset is used as the test set and the model was initialized. Consequently, all data from the 425 participants were inferred once. Each fold experiment commenced after completely resetting the previous learning results, and the performance metric represented the prediction outcomes for data not used in the learning process (**Figure 1**).

#### 2.4.5 Model performance indicators

In cases where machine learning is used to solve classification problems, accuracy can be used as a model evaluation metric, calculated as the ratio of correctly predicted samples to the total number of samples. True positives (TP) occur when a positive class is correctly classified as positive, false negatives (FN) when a positive class is incorrectly classified as negative, false positives (FP) when a negative class is incorrectly classified as positive, and true negatives (TN) when a negative class is correctly classified as negative. The accuracy metric is calculated as follows:


$$accuracy\;=\frac{TP\;+\;TN}{TP\;+\;FP\;+\;FN\;+\;TN}$$

The accuracy metric has the advantage of being intuitive and easy to interpret; however, it does not consider the differences between the classes being classified. The F1-score, or F1-measure, is widely used to address this limitation because it considers both precision and recall, and gives more weight to the lower value. This way, it penalizes models that perform well on some classes but poorly on others, promoting a more balanced performance. Precision is the ratio of correctly predicted positive observations to the total predicted positives. Recall is the ratio of correctly predicted positive observations to all observations in the actual positives. The F1 score is the harmonic mean of precision and recall.


$$precision\;=\frac{TP}{TP\;+\;FP}$$

$$recall\;=\;\frac{TP}{TP\;+\;FN}\;$$

$$F1\;score\;=\frac{2\;*\;precision\;*\;recall}{\;precision\;+\;recall}$$

In this study, we divided the neuroticism level into three classes (below-average, average, and above-average) and calculated the accuracy and F1 score. For multiclass classification, the precision and recall can be calculated by setting the value for one class as true and the values for the others as false. This process was repeated for each class. Consequently, one F1 score was calculated for each class, and the average of the scores was reported as the result (macro average).

#### 2.4.6 Comparison with human prediction accuracy

Given the absence of a predetermined benchmark for the model’s predictive performance, we adopted the comparative predictive accuracy of five postgraduate students majoring in clinical and counseling psychology. Out of a total of 425 participants, the response set (18 items) from 50 participants was randomly selected for human evaluation. Each rater was assigned to evaluate the responses of 10 participants, resulting in a total of 10 sets of 18 responses per rater. Since the model training process was conducted for each individual item rather than the entire response set, the responses to each question were provided separately and the order of responses was randomized to prevent inference using responses to other items. During the evaluation, raters were only provided with the text version of the responses and blinded to any other demographic, clinical data, or the result of prediction models. Raters inferred the level (below-average, average, and above-average) of neuroticism, dependency, and depressivity, and the mean accuracy for each item of each trait was calculated.

## 3 Results

### 3.1 Prediction performance

**Table 3** presents the items that were effective and ineffective in predicting neuroticism, respectively. The accuracy was 0.48 for the best-performing item (Item 7) and 0.41 for the poorest-performing item (Item 8). In terms of the F1 score, the average value was 0.39, with the best-performing item at 0.45 (Item 7) and the poorest-performing item at 0.34 (Item 3). The full neuroticism prediction performance for each item is presented in **Supplementary Table 2**.

**Table 3:** Neuroticism prediction.

Effective			Ineffective		
Item	Accuracy	F1	Item	Accuracy	F1
7. Do you frequently compare yourself to others? Please share about this.	0.48	0.45	8. How do you feel and think when you meet new kinds of experiences?	0.41	0.37
14. How do you feel about someone who may be unintentionally hurt or harmed by you?	0.46	0.43	16. Tell me if there is something unusual in your actions or thoughts, or something that other people do not understand well.	0.42	0.35
17. Please share your negative feelings or thoughts if you have any.	0.46	0.42	3. What is the main reason for having difficulties in trusting your friends or associates?	0.42	0.34

*n* = 128 (30%) below-average neuroticism, *n* = 134 (32%) average neuroticism, and *n* = 163 (38%) above-average neuroticism.

Considering both accuracy and F1 scores, the item that best predicted neuroticism was item 7 (“Do you frequently compare yourself to others? Please share this information.”). Compared with the other items, item 17 (“Please share your negative feelings or thoughts if you have any.”) and 14 (“How do you feel about someone who may be unintentionally hurt or harmed by you?”) also demonstrated superior performance. In contrast, items that exhibited poor performance in neuroticism prediction included item 3 (“What is the main reason for having difficulties trusting your friends or associates?”), item 8 (“How do you feel and think when you have new kinds of experiences?”), and item 16 (“Tell me if there is something unusual in your actions or thoughts, or something that other people do not understand well.”).

**Table 4** presents the items that were effective and ineffective in predicting depressivity. The accuracy was 0.49 for the best-performing item (Item 9) and 0.41 for the poorest-performing item (Item 8). In terms of the F1 score, the average value was 0.38, with the best-performing item at 0.47 (Item 17) and the poorest-performing item at 0.26 (Item 8 and 16). The full depressivity prediction performance for each item is presented in **Supplementary Table 3**.

**Table 4:** Depressivity prediction.

Effective			Ineffective		
Item	Accuracy	F1	Item	Accuracy	F1
17. Please share your negative feelings or thoughts if you have any.	0.47	0.47	8. How do you feel and think when you meet new kinds of experiences?	0.41	0.26
7. Do you frequently compare yourself to others? Please share about this.	0.48	0.45	12. To what extent do you want to be recognized or treated by people?	0.43	0.27
9. How would you feel if someone else noticed your feelings (joy, happiness, anxiety, anger, sadness)?	0.49	0.43	16. Tell me if there is something unusual in your actions or thoughts, or something that other people do not understand well.	0.42	0.26

*n* = 181 (42%) for below-average depressivity, *n* = 134 (32%) for average depressivity, and *n* = 110 (26%) for above-average depressivity.

Considering both accuracy and F1 score, the item with the best-predicted depressivity was item 17 (“Please share your negative feelings or thoughts if you have any.”). Compared with the other items, Item 7 (“Do you frequently compare yourself to others? Please share this information.”) and 9 (“How would you feel if someone else noticed your feelings?”) also demonstrated superior performance. In contrast, items that exhibited poor performance in depressivity prediction included item 8 (“How do you feel and think when you have new kinds of experiences?”), item 12 (“To what extent do you want to be recognized or treated by people?”), and item 16 (“Tell me if there is something unusual in your actions or thoughts, or something that other people do not understand well.”).

**Table 5** presents the items that were effective and ineffective in predicting dependency. The accuracy was 0.48 for the best-performing item (Item 14) and 0.42 for the poorest-performing item (Item 16). In terms of the F1 score, the average value was 0.31, with the best-performing item at 0.38 (Item 14) and the poorest-performing item at 0.25 (Item 15). The full dependency prediction performance for each item is presented in **Supplementary Table 4**.

**Table 5:** Dependency prediction.

Effective			Ineffective		
Item	Accuracy	F1	Item	Accuracy	F1
14. How do you feel about someone who may be unintentionally hurt or harmed by you?	0.48	0.38	15. What is the main reason for having difficulties in doing things efficiently?	0.43	0.25
2. Do you prefer a leading position in your work or interpersonal relationships? Or do you prefer to contribute from a less leading position?	0.46	0.35	16. Tell me if there is something unusual in your actions or thoughts, or something that other people do not understand well.	0.42	0.27
17. Please share your negative feelings or thoughts if you have any.	0.44	0.35	5. How do you usually handle tasks that need to be completed in a given schedule?	0.44	0.27

*n* = 135 (32%) with below-average dependency, *n* = 108 (25%) with average dependency, and *n* = 182 (43%) with above-average dependency.

Considering both accuracy and F1 score, the item that best-predicted dependency was item 14 (“How do you feel about someone who may be unintentionally hurt or harmed by you?”). Item 2 (“Do you prefer a leading position in your work or interpersonal relationships? Or do you prefer to contribute from a less leading position?”) also demonstrated superior performance compared with the other items. In contrast, items that exhibited poor performance in dependency prediction included item 15 (“What is the main reason for having difficulties doing things efficiently?”) and item 16 (“Tell me if there is something unusual in your actions or thoughts, or something that other people do not understand well.”).

### 3.2 Comparison with human prediction accuracy

On average, clinical psychology graduate students reported an accuracy of 0.47 for neuroticism, 0.51 for depressivity, and 0.38 for dependency. For the accuracy of each item, please refer to **Supplementary Table 5**. The overall predictive accuracy exhibited by the language-based prediction model was comparable to that of the graduate students. For the prediction of depressive levels, human evaluators demonstrated superior predictive accuracy compared to that of the language-based model, whereas the accuracy was similar in the case of neuroticism. Conversely, the model reported superior predictive accuracy for dependency.

## 4 Discussion

This study developed an LPA model predicting neuroticism and its facets by utilizing open-ended questions based on FFM and NLP techniques. Previous studies with pre-existing social media data barely provided guideline for applying LPA in practical settings. However, exploring appropriate questions for LPA is particularly important in practical settings because questions determine the context of elicited responses. This study examined the model’s accuracy and the influence of item content in predicting neuroticism.

The models demonstrated a level of predictive accuracy comparable with that of graduate students majoring in clinical psychology. The overall predictive performance was consistent with the findings of previous studies. Considering the difficult task of classifying neuroticism levels into three classes and that no previous studies have used a multi-class classification approach, this study’s predictive accuracy appeared to be at an appropriate level compared to recent personality prediction studies (including studies by Ghosh et al. (52) with F1 scores ranging from 0.36 to 0.54, Yang et al. (53) with F1 scores ranging from 0.59 to 0.71, and Christian et al. (12) with an F1 score of 0.69 for the neuroticism binary classification task).

Additionally, this study identified effective questions for predicting neuroticism. Although all open-ended items were developed for measuring personality, asking about social comparisons or directly inquiring about negative emotions and thoughts proved to be effective in accurately predicting overall neuroticism. Intriguingly, item 7 was considered potentially relevant to neuroticism by external expert committees, and item 17 was initially formulated to target neuroticism. However, items intended to measure other traits such as openness, psychoticism, and agreeableness, which theoretically have weak connections to neuroticism, consistently performed poorly in predicting neuroticism and its facets of depressivity and dependency.

The approach adopted in this study, utilizing open-ended questions, effectively addresses the limitations of previous research while linking predictive results to psychological meaning. Primarily, because the open-ended questions were developed by psychologists based on personality theories, they serve as a framework ensuring that responses are relevant to personality traits. For psychologists, the process of developing and validating questions that accurately reflect the constructs being measured is a familiar one, and through this process, the items typically attain content validity. While the field of psychology has made efforts to address issues of high face validity (54, 55), in the context of machine learning, concerns regarding content validity and construct irrelevance have emerged. In this situation, open-ended questions grounded in personality theory play a crucial role in ensuring that the responses collected are pertinent to personality.

Another significant contribution of the current study is that it allows for examining whether the content of the items influences the predictive accuracy. The theoretical role and discriminant validity aspects have often been overlooked in the field of personality assessment using computational science or machine learning. In this study, prediction models generated using items designed to measure neuroticism consistently showed superior performance, while models created using items intended to measure constructs less related to neuroticism, such as openness or psychoticism, consistently showed poorer performance. Utilizing data closely related to the construct being predicted is not only theoretically significant but also enhances the predictive power of the model. While it may seem intuitive, this has thus far only been mentioned at the theoretical level in the field of CPA and is yet to be empirically investigated (9, 17).

On the other hand, it is also worth discussing items that were not designed for measuring neuroticism but proved effective in predicting neuroticism, such as item 7. Although we expected the question regarding social comparison to be associated with neuroticism, the target variable assigned to this question was antagonism. However, item 7, which asked respondents to provide information about social comparison, was the most effective question for predicting neuroticism. This may be consistent with previous studies, where predictions introduced novel hypotheses in directions unanticipated by existing theories (56, 57). Social comparison has not traditionally been considered a core factor of neuroticism in personality theories, but data-driven approach can offer novel perspectives. Recent studies have actively investigated the association between social comparison and emotions such as depression, anxiety, feelings of inferiority, and envy (58–60). Inquiring about social comparison can be a useful approach to gather information about a client’s neuroticism trait and potential mental health issues, while mitigating face validity concerns.

A key limitation of this study is the small dataset size, leaving room for potential improvements in the BERT-based personality prediction model. Using newly collected data rather than pre-existing data made it difficult to obtain a large dataset. While previous studies that collected new dataset have used similar dataset sizes (61, 62), the current sample is still small compared to most natural language models, necessitating a larger dataset for better performance. Thus, the current results should be seen as preliminary, useful for comparing the relative performance of each item, but not indicative of the full potential of natural language-based multiclass personality classification. The limited data size also prevented us from separating training and validation data, and led us to choose a classification approach over a regression approach. Future research should secure a larger dataset for higher quality training and validation, possibly enabling better performance with a regression approach and separate validation set. Validating the current model with data from an independent cohort could also be promising. The current result may aid collection of larger dataset, helping to identify the key questions among full 18 items.

Another limitation is that, although this study identified questions that were effective or ineffective in predicting neuroticism, a deeper exploration is required to explain this outcome. For example, we were unable to clearly interpret the predictive performance of item 18. This item was developed to measure neuroticism; however, item 18 demonstrated only a modest level of predictive performance for neuroticism. Expert judgment, not quantification, links each item’s content to neuroticism, which we used as an explanatory basis. Future studies should develop methods to quantify, explain and interpret the variance in performance attributable to item content. Traditional language analysis such as lexical analysis can be adopted to provide more deep understanding the relationship between language data and personality (20). Lastly, one of the advantages of the current approach is that open-ended questions may have an edge over traditional Likert self-report questionnaires in mitigating impression management and deceit. However, responses to open-ended questions are still self-reported, and the predictive performance of neuroticism levels was evaluated based on self-report questionnaires. Future research should consider integrating more objective personality reports when training or validating the prediction model, and work towards reducing the impact of response biases and impression management.

In conclusion, our study presents a paradigm that can conduct language-based analyses based on the relatively small sample sizes typically available in psychology, demonstrating meaningful implications for CPA and personality psychology. This study is the first to demonstrate that open-ended questions based on personality theory can be adopted as prompts for predicting personality traits using NLP. For a CPA to be an effective alternative to traditional self-reported questionnaires, it should demonstrate adequate psychometric validity, including reliability, discriminant validity, and content validity. This study paves the way for examining the influence of item content on prediction models. The study also offers practical guidelines for language-based personality assessments by adopting open-ended questions based on a five-factor personality model. Computational personality research can advance our understanding of human personalities through the integration of top-down and data-driven approaches.

## Data availability statement

The raw data supporting the conclusions of this article will be made available by the authors for qualified researchers, without undue reservation.

## Ethics statement

The studies involving humans were approved by Korea University Institutional Review Board. The studies were conducted in accordance with the local legislation and institutional requirements. The participants provided their written informed consent to participate in this study.

## Author contributions

SY: Writing – original draft, Project administration, Methodology, Investigation, Data curation, Conceptualization. JJ: Writing – review & editing, Methodology, Investigation, Conceptualization. GS: Writing – review & editing, Methodology, Investigation. SP: Writing – review & editing, Methodology, Investigation. JH: Writing – review & editing, Methodology, Investigation. JC: Writing – review & editing, Supervision, Software, Methodology, Funding acquisition, Formal analysis, Conceptualization. KC: Writing – review & editing, Supervision, Project administration, Methodology, Funding acquisition, Conceptualization.
