# Supplementary material for: Predicting neuroticism with open-ended response using natural language processing
Source: Front Psychiatry. 2024 Aug 1;15:1437569. doi: 10.3389/fpsyt.2024.1437569 (PMC11324482; doi:10.3389/fpsyt.2024.1437569)
Supplement: Supplementary file 1 [file Table_1.docx]

Supplementary Material

**Supplementary Table 1.** Corresponding BDPI domains for each items

|  | Questions | Domain |
| --- | --- | --- |
| 1 | How do you want to spend your time for your routine daily hours? | Extraversion |
| 2 | Do you prefer a leading position in your work or interpersonal relationships? Or do you prefer to contribute from a less leading position? | Extraversion |
| 3 | What is the main reason for having difficulties in trusting your friends or associates? | Agreeableness |
| 4 | What do you usually think and do when someone asks for help? Tell us how you feel and think when rejecting someone's request | Agreeableness |
| 5 | How do you usually handle tasks that need to be completed in a given schedule? | Conscientiousness |
| 6 | To what extent do you tend to achieve the standards or goals you set for yourself? | Conscientiousness |
| 7 | Do you frequently compare yourself to others? Please share about this. | Egocentrism |
| 8 | How do you feel and think when you meet new kinds of experiences? | Openness |
| 9 | How would you feel if someone else noticed your feelings (joy, happiness, anxiety, anger, sadness)? | Emotional  stability |
| 10 | To what extent do you feel cautious or want to avoid interpersonal relationships? | Detachment |
| 11 | How close do you want your relationships to be with others? | Detachment |
| 12 | To what extent do you want to be recognized or treated by people? | Egocentrism |
| 13 | How do you feel about being noticed or receiving attention from others? | Egocentrism |
| 14 | How do you feel about someone who may be unintentionally hurt or harmed by you? | Egocentrism |
| 15 | What is the main reason for having difficulties in doing things efficiently? | Disinhibition |
| 16 | Tell me if there is something unusual in your actions or thoughts, or something that other people do not understand well. | Psychoticism |
| 17 | Please share your negative feelings or thoughts if you have any. | Negative  affectivity |
| 18 | Are you spontaneous and highly influenced by your mood? What do you do when you feel negative emotions? | Negative  affectivity |

**Supplementary Table 2.** Neuroticism prediction performance of each item

|  | Accuracy | F1 score | Precision | Recall |
| --- | --- | --- | --- | --- |
| Item 1 | 0.45 | 0.38 | 0.40 | 0.43 |
| Item 2 | 0.44 | 0.41 | 0.43 | 0.42 |
| Item 3 | 0.42 | 0.34 | 0.41 | 0.41 |
| Item 4 | 0.45 | 0.41 | 0.42 | 0.44 |
| Item 5 | 0.44 | 0.36 | 0.37 | 0.42 |
| Item 6 | 0.44 | 0.39 | 0.41 | 0.42 |
| **Item 7** | **0.48** | **0.45** | **0.48** | **0.48** |
| Item 8 | 0.41 | 0.37 | 0.38 | 0.39 |
| Item 9 | 0.48 | 0.35 | 0.40 | 0.42 |
| Item 10 | 0.44 | 0.39 | 0.42 | 0.43 |
| Item 11 | 0.45 | 0.39 | 0.48 | 0.43 |
| Item 12 | 0.46 | 0.43 | 0.45 | 0.44 |
| Item 13 | 0.45 | 0.38 | 0.39 | 0.44 |
| Item 14 | 0.46 | 0.43 | 0.45 | 0.44 |
| Item 15 | 0.45 | 0.42 | 0.44 | 0.44 |
| Item 16 | 0.42 | 0.35 | 0.41 | 0.40 |
| Item 17 | 0.46 | 0.42 | 0.45 | 0.45 |
| Item 18 | 0.43 | 0.39 | 0.44 | 0.42 |

*Note:* *n* = 128 (30%) below-average neuroticism, *n* = 134 (32%) average neuroticism, and *n* = 163 (38%) above-average neuroticism; Bolded row indicates the best-performing item

**Supplementary Table 3.** Depressivity prediction performance of each item

|  | Accuracy | F1 score | Precision | Recall |
| --- | --- | --- | --- | --- |
| Item 1 | 0.46 | 0.42 | 0.45 | 0.43 |
| Item 2 | 0.46 | 0.39 | 0.46 | 0.42 |
| Item 3 | 0.44 | 0.42 | 0.45 | 0.43 |
| Item 4 | 0.46 | 0.42 | 0.46 | 0.43 |
| Item 5 | 0.44 | 0.33 | 0.41 | 0.37 |
| Item 6 | 0.43 | 0.34 | 0.34 | 0.38 |
| Item 7 | 0.48 | 0.45 | 0.46 | 0.46 |
| Item 8 | 0.41 | 0.26 | 0.29 | 0.34 |
| Item 9 | 0.49 | 0.43 | 0.56 | 0.46 |
| Item 10 | 0.44 | 0.40 | 0.42 | 0.42 |
| Item 11 | 0.46 | 0.31 | 0.40 | 0.38 |
| Item 12 | 0.43 | 0.27 | 0.27 | 0.35 |
| Item 13 | 0.46 | 0.43 | 0.45 | 0.44 |
| Item 14 | 0.45 | 0.29 | 0.40 | 0.37 |
| Item 15 | 0.46 | 0.44 | 0.46 | 0.44 |
| Item 16 | 0.42 | 0.26 | 0.28 | 0.34 |
| **Item 17** | **0.47** | **0.47** | **0.51** | **0.47** |
| Item 18 | 0.45 | 0.41 | 0.44 | 0.42 |

*Note:* *n* = 181 (42%) for below-average depressivity, *n* = 134 (32%) for average depressivity, and *n* = 110 (26%) for above-average depressivity; Bolded row indicates the best-performing item

**Supplementary Table 4.** Dependency prediction performance of each item

|  | Accuracy | F1 score | Precision | Recall |
| --- | --- | --- | --- | --- |
| Item 1 | 0.44 | 0.32 | 0.35 | 0.39 |
| Item 2 | 0.46 | 0.35 | 0.35 | 0.40 |
| Item 3 | 0.43 | 0.36 | 0.39 | 0.38 |
| Item 4 | 0.45 | 0.32 | 0.33 | 0.39 |
| Item 5 | 0.44 | 0.27 | 0.31 | 0.36 |
| Item 6 | 0.45 | 0.32 | 0.32 | 0.38 |
| Item 7 | 0.44 | 0.30 | 0.29 | 0.37 |
| Item 8 | 0.45 | 0.30 | 0.33 | 0.37 |
| Item 9 | 0.44 | 0.27 | 0.29 | 0.36 |
| Item 10 | 0.46 | 0.31 | 0.38 | 0.38 |
| Item 11 | 0.41 | 0.29 | 0.27 | 0.36 |
| Item 12 | 0.44 | 0.34 | 0.39 | 0.39 |
| Item 13 | 0.45 | 0.30 | 0.36 | 0.37 |
| **Item 14** | **0.48** | **0.38** | **0.41** | **0.42** |
| Item 15 | 0.43 | 0.25 | 0.36 | 0.34 |
| Item 16 | 0.42 | 0.27 | 0.27 | 0.34 |
| Item 17 | 0.44 | 0.35 | 0.37 | 0.40 |
| Item 18 | 0.44 | 0.31 | 0.37 | 0.37 |

*Note:* *n* = 135 (32%) with below-average dependency, *n* = 108 (25%) with average dependency, and *n* = 182 (43%) with above-average dependency; Bolded row indicates the best-performing item

**Supplementary Table 5.** Accuracy of human prediction

|  | Neuroticism | Dependency | Depressivity |
| --- | --- | --- | --- |
| Item 1 | 0.43 | 0.30 | 0.23 |
| Item 2 | 0.37 | 0.40 | 0.37 |
| Item 3 | 0.47 | 0.27 | 0.33 |
| Item 4 | **0.60** | 0.37 | 0.30 |
| Item 5 | 0.43 | 0.47 | 0.33 |
| Item 6 | 0.40 | **0.50** | 0.37 |
| Item 7 | 0.57 | 0.33 | 0.37 |
| Item 8 | 0.53 | 0.40 | 0.30 |
| Item 9 | 0.37 | 0.37 | 0.33 |
| Item 10 | **0.60** | 0.33 | 0.30 |
| Item 11 | 0.40 | 0.47 | 0.37 |
| Item 12 | 0.33 | 0.30 | 0.23 |
| Item 13 | 0.47 | 0.40 | 0.30 |
| Item 14 | 0.47 | 0.40 | 0.27 |
| Item 15 | 0.43 | 0.30 | 0.23 |
| Item 16 | 0.40 | 0.33 | 0.33 |
| Item 17 | 0.53 | 0.43 | 0.40 |
| Item 18 | 0.53 | 0.37 | **0.43** |

*Note.* accuracy, 3-class classification; The best performances are bolded
